# Truth and Error

**Published:** 1870-02

**Authors:** 


### TRUTH AND ERROR.

Truth being founded on a rock, you must boldly dig to see its foundations without fear of destroying the edifice ; but falsehood on the sand, if you examine its foundations, you cause its fall.

